# Supplementary figures and images for: Memory phase-specific genes in the Mushroom Bodies identified using CrebB-target DamID
Source: PLoS Genet. 2023 Jun 12;19(6):e1010802. doi: 10.1371/journal.pgen.1010802 (PMC10289670; doi:10.1371/journal.pgen.1010802)

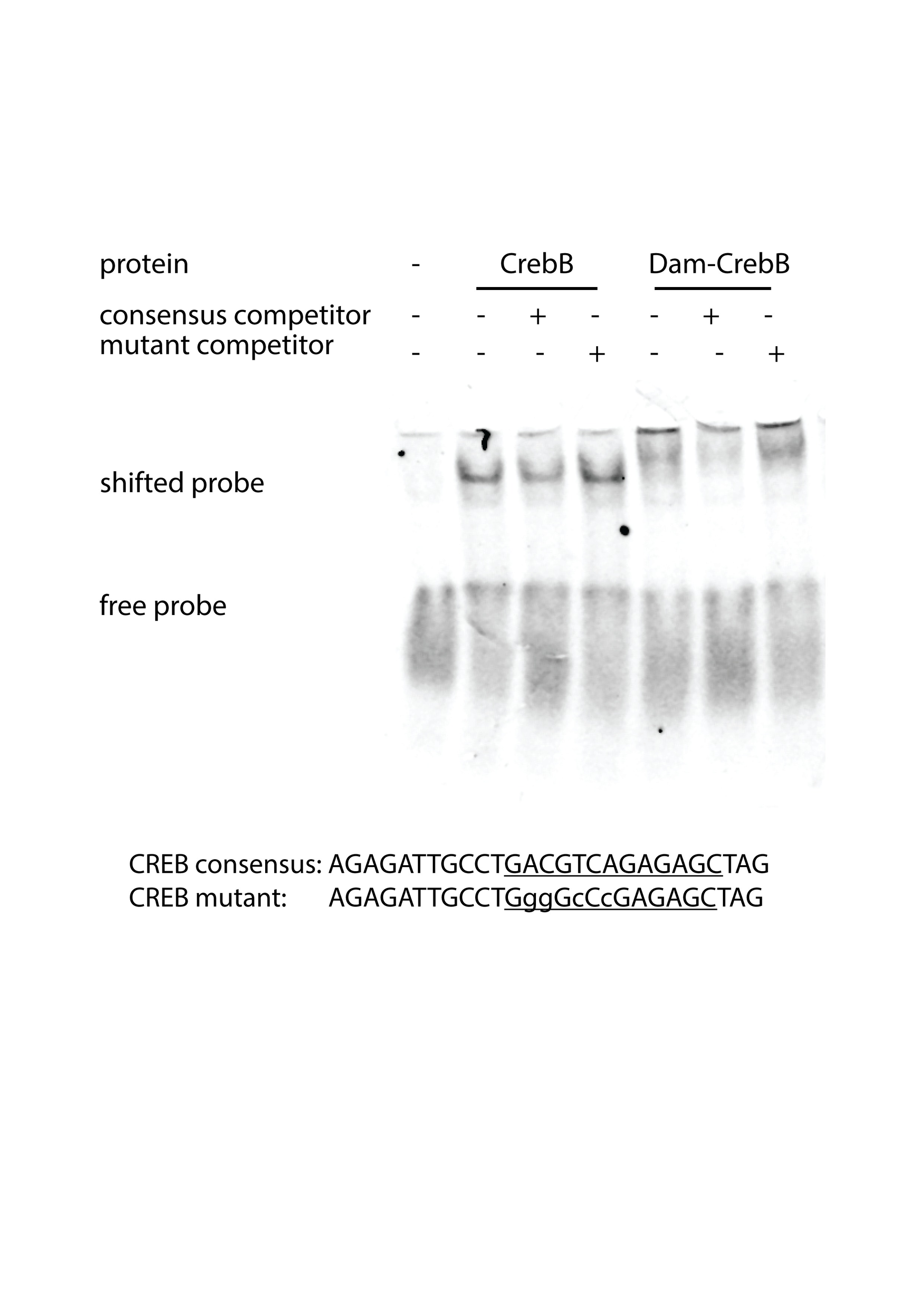

Supplement: S1 Fig — Electrophoretic Mobility Shifts for CrebB and Dam-CrebB binding to CREB-site. Labeled probe is bound by CrebB protein alone and by Dam::CrebB fusion protein resulting in a shift of the signal (shifted probe). Addition of unlabeled oligonucleotides with the same sequence as the probe (consensus competitor) reduces the amount of shifted probe, while addition of unlabeled oligonucleotides with a mutated binding site (mutant competitor) does not reduce the amount of shifted probe. Below the gel the sequences of the labeled probe and consensus competitor oligonucleotide (CREB consensus) and the mutant competitor oligonucleotide (CREB mutant) are shown. Mutated residues in small letters. Binding site underlined. (TIF) [file pgen.1010802.s001.tif]

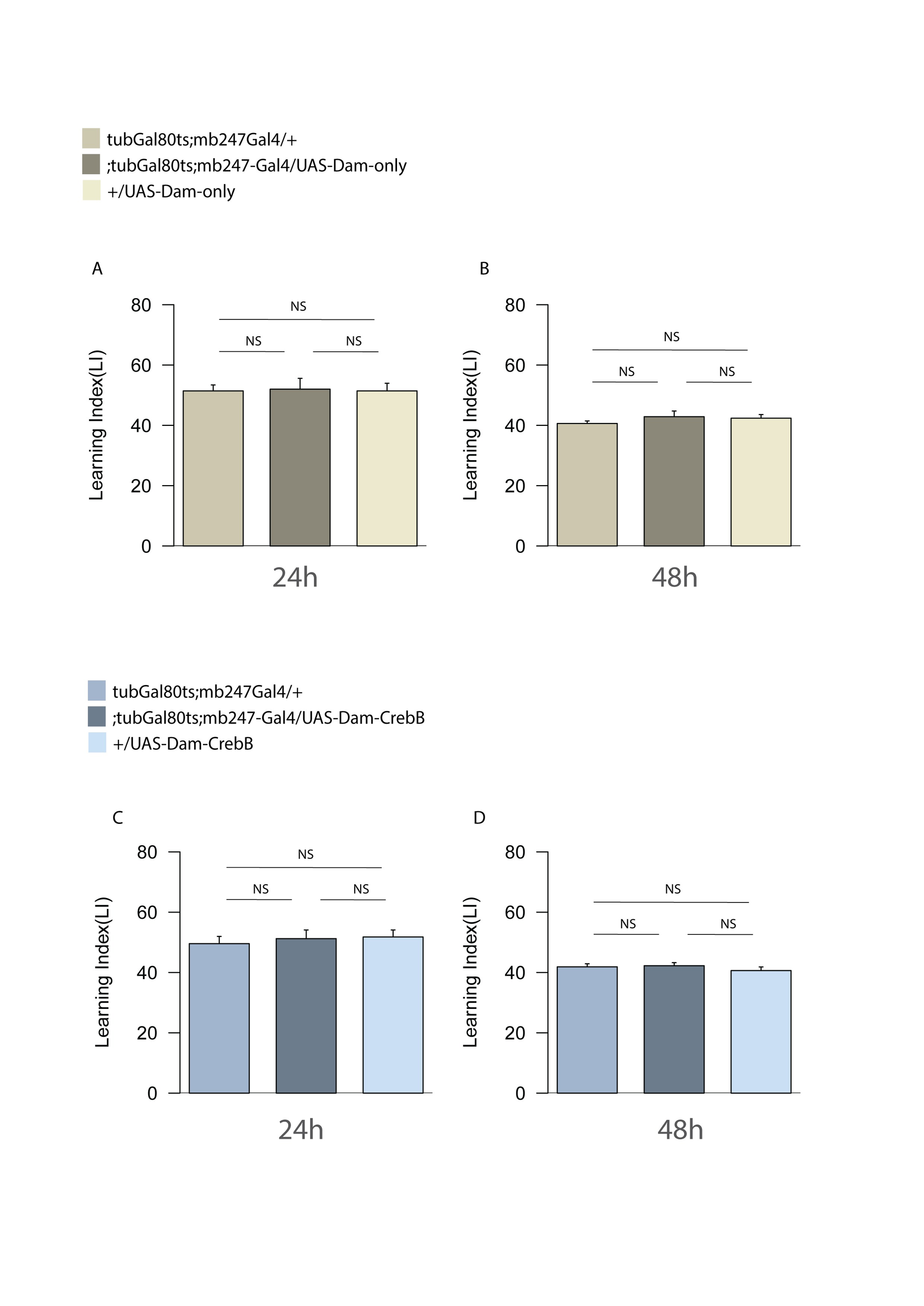

Supplement: S2 Fig — UAS-Dam-only or UAS-Dam::CrebB expression was induced in the MB of adult flies using the driver mb247-Gal4 and co-expressing tub-Gal80ts. Transgenic crosses set up at the permissive temperature (18°C) and adult flies were transferred to 29°C for3/4 days. Adult flies expressing Dam-only did not show changes in the 24h or 48h memory performance (A-B). Similarly, adult flies expressing Dam::CrebB did not show differential learning scores at the same time points (C-D). Asterisks indicate the P-value of the comparison with control crosses (mb247-Gal4>w1118/ y1w1/y1v1/y1sc*v1sev21) (* P < 0.05, ** P < 0.01, *** P < 0.001); n = 7–10 for MB > RNAi, n = 7–28 for control crosses. Bar graphs represent the mean and error bars represent the SEM. (TIF) [file pgen.1010802.s002.tif]

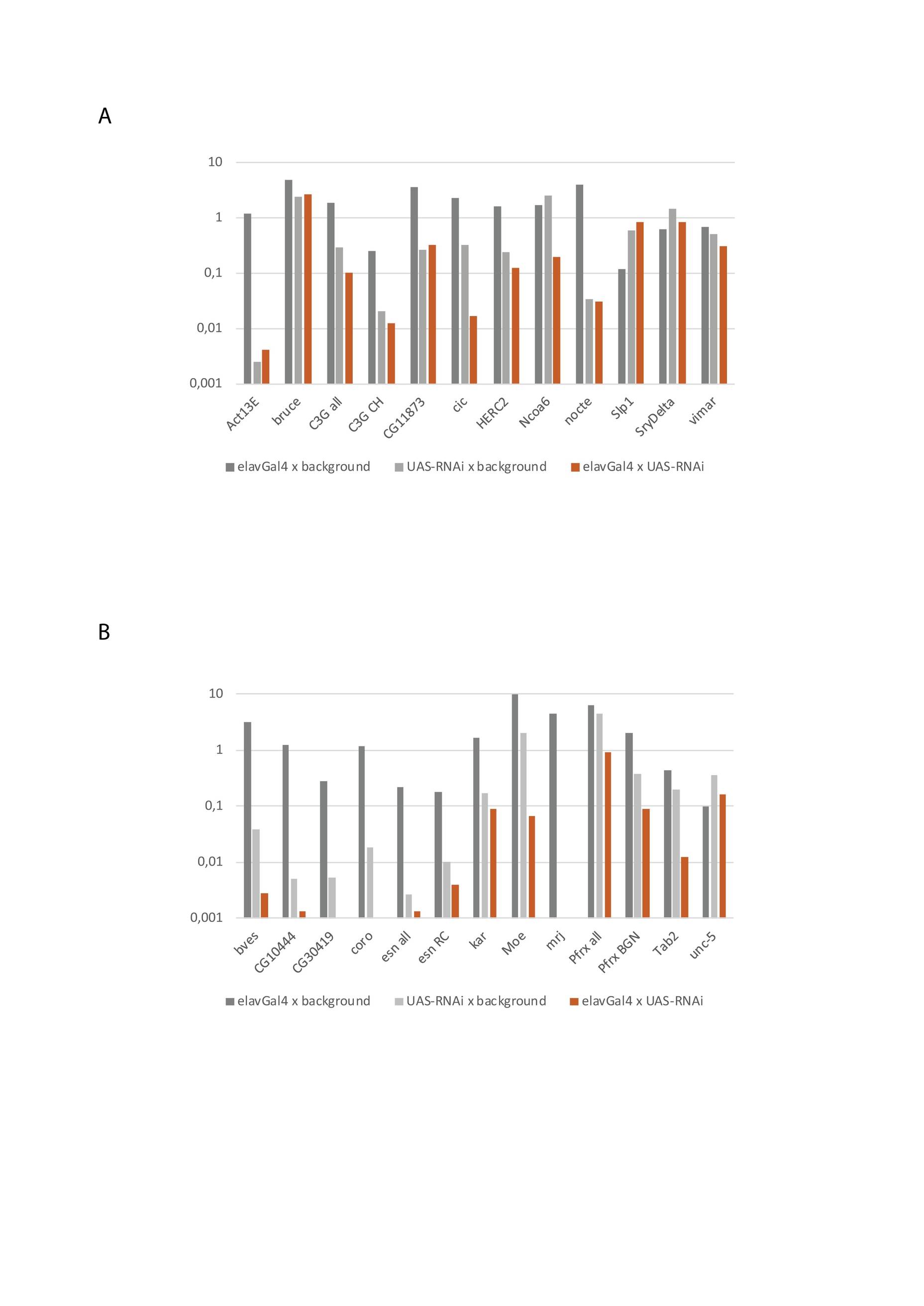

Supplement: S3 Fig — qPCR on RNAi-lines crossed to elav-Gal4 (orange bars) to analyze RNAi-levels in comparison to controls (elav-Gal4 x background, dark grey; and UAS-RNAi lines x background, light grey; expression levels relative to endogenous actin42C RNA levels, arbitrary units). (TIF) [file pgen.1010802.s003.tif]

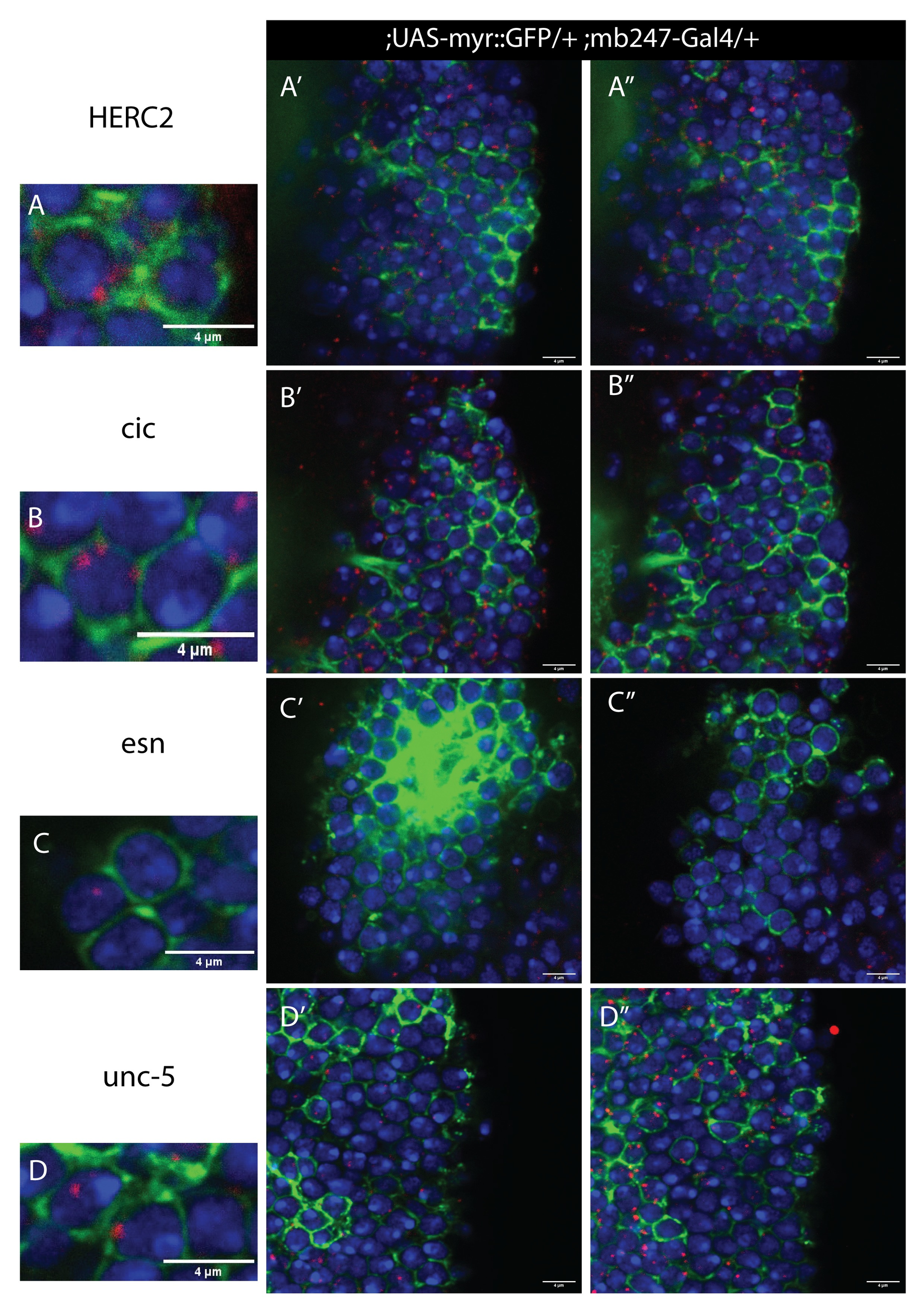

Supplement: S4 Fig — (A, B, C, D) magnified images of MB cells, showing the mRNA expression of the 4 hits. (A’, A”) alternative sections of HERC2 mRNA expression (red). (B’,B”) alternative sections of cic mRNA expression (red). (C’,C”) alternative sections of esn mRNA expression (red). (D’,D”) alternative sections of unc-5 mRNA expression (red). mb247>GFP (green), Dapi (blue). (TIF) [file pgen.1010802.s004.tif]
