# Supplementary material for: Memory phase-specific genes in the Mushroom Bodies identified using CrebB-target DamID
Source: PLoS Genet. 2023 Jun 12;19(6):e1010802. doi: 10.1371/journal.pgen.1010802 (PMC10289670; doi:10.1371/journal.pgen.1010802)
Supplement: Attachment — Submitted filename: RESPONSE_TO_REVIEWERS.pdf [file pgen.1010802.s013.pdf]

Comments to the Authors:

Please note here if the review is uploaded as an attachment.

Reviewer #1: In this work, Sgammeglia et al generate UAS-Dam::CrebB tool for identifying CrebB target gene expressions in olfactory memory center, the mushroom body, after appetitive conditioning. Combined with tub-GAL80ts transgene allows the authors to distinguish the CrebB target gene expressions within different time intervals (TIs) via shifting the flies from permissive to restrictive temperature during memory formation. Two different TIs protocols were used in their assays, which are 3-hour before training to 24-hour after training(TI-1) and 24 to 48 hour after training (TI-2). The authors also compared with previous ChIP-seq dataset and identified 16 and 17 genes are differentially regulated in TI-1 and TI-2, respectively. RNA interference (RNAi)-mediated gene silencing and behavioral screen identified 4 genes as potential targets of CrebB. Genetic silencing of these 4 genes in mushroom body also disrupted appetitive memories. Overall, this study provide an elegant tool for investigating potential genes under CrebB regulations in specific neurons. However, there are several issues are needed to be resolved before publication. Listed below are my specific comments:

Major comments:

1. The major contribution of this study is based on the Dam::CrebB fusion protein, which allows for identifying the potential CrebB target gene expression in specific neurons (this study focused on mushroom body). However, the effects of genetic modification of CrebB was not checked before their experiments. Whether the Dam::CrebB fusion protein still can bind to CRE as regular CrebB does? The authors should designed experiments to show that CRE binding ability is not affected in the Dam::CrebB as compared to regular CrebB.

Thank you for pointing this out. We have now cloned the Dam::CrebB and CrebB coding sequences into the pBluescript vector to express the proteins in vitro. With those we performed a gel-shift experiment in which we tested for binding of both protein versions to a commercially available labeled DNA-oligo containing the consensus CRE sequence. To further verify the binding specificity, we tested binding in the presence of either a specific unlabeled competitor oligo consisting of the same sequence as the labeled oligo or of an unlabeled oligo in which the CRE sequence is mutated. The results of this experiment show that Dam::CrebB binds specifically to the CRE site as does CrebB alone. We now included this experiment in supplementary Figure S1 to the manuscript.

2. The experimental design for identifying the candidate CrebB downstream gene expression after paired training includes 2 time intervals (TI-1 and TI-2 in Figure 1B), which shift the temperature from 18 to 29 °C at different periods for silencing GAL80ts activity. However, the permissive temperature condition should be included in this experiments, which is the same flies all the way stay in 18 °C.

Thanks for this comment. We performed LTM tests at 24h and 48h for UAS-Dam::CrebB and UAS-Dam, respectively, in experimental and control crosses raised at the permissive temperature, to evaluate whether the temperature shift included in the experiment was affecting LTM formation and retention. The learning results are now

included in the manuscript as supplementary Figure S2. The temperature shifts do not affect learning.

3. A lot of UAS-geneRNAi lines target different genes were used in this study, but there is no any verification for the knockdown efficiency of the RNAi lines used in this study.

We appreciate this feedback. To validate the knockdown efficiency, we have performed a qPCR analysis in whole fly heads, pan-neuronally knocked down for each gene (Elav-Gal4 x UAS-RNAi), and compared to the controls (Elav-Gal4/+ and +/UAS-RNAi). Of the 22 genes tested 3 were lethal with the elav-Gal4 driver, 13 have shown reduced mRNA expression compared to their controls. The remaining 6 genes did not show a reduction compared to both controls, probably due to inefficient knockdown, or a higher expression in those cells that were not addressed by our pan-neuronal Gal-4 line (e.g. glial cells). In many cases the +/UAS-RNAi control already shows a reduction of RNA levels compared to the Elav-Gal4 control suggesting that there is some leaky expression of these UAS-RNAi constructs. The results of the qPCR screen are shown in the supplementary Figure S3.

4. Insufficient controls in several figures. All the behavioral data of the manuscript only use GAL4/+ as the genetic control groups but the UAS-geneRNAi/+ controls are missing in whole manuscript. The UAS-geneRNAi/+ control groups should also be conducted and included in the behavioral data.

Many thanks for this important observation. We admit that the UAS-geneRNAi/+ experiment is an important control. For each gene of TI-1 and TI-2, we performed 24h and 48 LTM tests, respectively, of their UAS-RNAi/+ control. As compared to the Gal4/+ control, *HERC2*, *esn*, *cic* and *unc-5* MB-knockdown showed a learning phenotype also in comparison to their UAS-geneRNAi/+ controls. We updated the Figure 2, which now includes all the controls.

5. Since unc-5 is important for the development neurites, therefore RNAi-mediated silencing of unc-5 in mushroom body affects memory may cause by the developmental defects of mushroom body. The authors should combine gene switch tools in their assay (i.e. tub-GAL80ts or MB-GeneSwitch) for bypassing the developmental effects.

Thanks, indeed restricting the knockdown to the adult stage is a straightforward way to filter out the developmental implication of unc-5. By co-expressing tub-Gal80ts in the MB, we could test unc-5 RNAi in adult flies, as reported in the new Figure 4. We extended the analysis also to the other 3 hits (*HERC2*, *cic* and *esn*). The results from these LTM tests were in line with our previous observation.

6. In Figure 3D, knockdown of esn in mushroom body disrupted The 24- and 48- hour appetitive memories but the esn mRNA is not detectable in mushroom body neurons. The authors should use other techniques (i.e. immunohistochemistry) instead of in situ hybridization to detect the expression of esn in mushroom body.

We thank the reviewer for pointing this out. Both HCR and single cell expression have shown expression of esn in only a small number of MB cells. A possible explanation for this may be that esn expression is only transiently induced during LTM formation. In this case it would be only detectable in 1-2 cells in the 1-2 day interval after the training. This may not be detectable with any of the methods. However, the fact that we observe an LTM increase upon esn knock-down in the mushroom body suggests that esn does have a function in LTM formation.

Minor comments:

1. line 62-62, the it should be: αβ and α'β' KCs projections form vertical and horizontal lobes, whereas γ KCs only form horizontal lobe of MB.

Thanks for this correction, the text is now updated.

Reviewer #2: Long-term appetitive memory requires CREB-dependent de novo gene expression and protein synthesis. Sgammeglia et al. developed the TaDa technique to identify CerebB target candidate genes during two-time intervals; TI-1 (0~24 hrs after training) and TI-2 (24 ~ 48 hrs after training). The authors assigned TI-1 for memory formation and consolidation and TI-2 for maintenance and found 111 genes in TI-1 and 26 genes in TI-2. Among candidate genes, the authors found that knocking down HERC2 and cic (TI-1) and esn and unc-5 (TI-2) impair memory.

While the authors developed a new gene screening method, due to practical concerns, they do not show how these genes are involved in long-term memory. For example, while they claimed that cic is the TI-1 gene and unc-5 is the TI-2 genes, knockdown of these genes resulted in defects in 0-, 24-, and 48 hr-memories. Therefore, it is unclear whether the functions of these genes are required during the assigned time interval, and whether their expressions are changed.

Major concerns

1. Temporal gene knockdown. It is unclear whether observed memory defects are caused by acute dysfunction of candidate genes or developmental defects. Authors should knock down candidate genes at least at the adult stage using temporal knockdown methods, such as TARGET and Gene Switch system. As mentioned above, 0 hr memory impairment in cic and unc-5 mutants could be due to defects in neural development. Furthermore, while authors exclude RNAi lines that have problems with the viability and health of MB>RNAi offspring, temporal knockdown may resolve these issues and increase the number of genes related to LTM.

We thank the reviewer for this valuable point. Restricting the knockdown to the adult stage is indeed an efficient approach to exclude any eventual developmental implications of the candidate genes. By co-expressing tub-Gal80ts in the MB we tested the RNAi in adult flies for *Herc*, *esn*, *unc-5* and *cic*, as reported in the new Figure 4. The results obtained from these LTM tests were in line with our previous observation and showed that the observed memory phenotype is connected to an acute dysfunction of the candidate genes.

2. Genomic background. UAS-RNAi and Gal4 driver lines should be outcrossed with their UAS-RNAi parental control lines. The authors compared the memory of knockdown lines with their parental background lines. However, the reviewer thinks it is not sufficient. Every line in the lab stock has spontaneous mutations over time. Therefore,

the genetic background of the parental line in the stock center is likely to be different from the author's lab.

Many thanks for this comment, which indeed pointed out a valid aspect. We agree with the reviewer that, to obtain the most accurate knockdown assessment, outcrossing UAS and Gal4 lines with the UAS-RNAi parental control lines would have been the ideal strategy, for each knockdown. However, in the view of a screen with several UAS-RNAi lines, we opted for background controls, in order to organize our candidate genes list into groups. We now extended the experiments including the UAS-RNAi/+ control, in Figure 2.

3. Absence of quantification of transcripts. The authors did not attempt to examine whether TI-1 and TI-2 genes actually changed their expression during these time intervals upon LTM formation.

This is an interesting point. A technical limitation to assess this question is that only a low number of cells within the MB represent the actual engram cells. Therefore, LTM-related changes are likely occurring just in those cells. We currently lack genetic tools to uniquely FACS isolate these engram cells. To assess the relative expression levels of candidate genes and to test the efficacy of the knockdown pan-neuronally we performed qPCR tests in whole heads independent of training. The results of the qPCR screen are shown in the supplementary Figure S3.

Minor comments

Functional classification and annotation of 111 TI-1 and 26 TI-2 genes would be more informative for people in the research field.

Thanks for this suggestion. We have included the functional annotation to the Supplementary File_1.

Reviewer #3: This manuscript uses a CREB-DAM fusion followed by genetic screening to identify novel regulators of long-term memory. Long term olfactory memory is model that has received enormous attention, with the foundational studies showing fundamental differences between short and long-term memory taking place nearly 30 years ago. While the role of CREB is LTM is now known to be deeply evolutionarily conserved, the role of factors downstream of CREB is still incomplete. The olfactory training experiments are well controlled, allowing for comparison of CREB-DAM results from trained and untrained flies. Overall, the approach is novel and identifies multiple new genes that are involved in memory. The experiments are well controlled and it is a significant advance for the field. Additional mechanistic follow-up would increase the impact of this manuscript, though I do not believe it is essential for the initial description of the screen. Overall this is a strong manuscript on a topic that will be of broad interest to the community and the new genes identified provide a foundation for future studies.

1. The experimental protocol overexpresses CREBB. It's an important control to show that in the contexts of these manipulations (including temperature shifts) there is no effect on memory.

Thank you for this comment. Actually, due to the dicistronic nature of the UAS-mCherry-Dam-CrebB transgene, the Dam-CrebB fusion protein is expressed at very low levels. However, to add another control we now tested the memory performance of the TaDa experimental crosses and controls [tub-Gal80ts;mb247-Gal4/UAS-CrebB::Dam or Dam(only); tub-Gal80ts;mb247-Gal4/+; +/UAS-CrebB::Dam or Dam(only)], both after temperature shift for T1 and T2. The result of these experiments show no changes in memory performance. We have now included these results in Supplementary Figure S2.

2. A general point worthy of more discussion is the choice of MB-driver. In addition, these memories may be encoded sparsely in Kenyon cells, which may dilute the impact of the technique. This does not invalidate the results in any way, but is worthy of discussion.

This point is, indeed, worthy of discussion and we thank the reviewer for having mentioned it. As the engram cells among the KCs are very few, the use of a general MB-Gal4 driver may constitute a limitation in our study. We updated the manuscript to discuss this aspect further.

3. Line 164: It is surprising that health RNAi knockdown flies is an issue since this brain region is non-essential. This is likely due to non-specific expression of the driver. Is it possible to test with another driver?

This is a good point. To target the mushroom bodies we chose the driver mb247-Gal4 instead of the broader (and therefore potentially more lethal) OK107-Gal4 driver.

The genes that were excluded, however, were not all lethal. The selection took into account especially whether the number of offspring was sufficient for LTM training/test.

4. The HCR data in Figure 3 is insufficient. Perhaps magnified images of individual cells would more clearly highlight the localization? One drawback is only a fraction of the MB cells are shown. Is it possible to predict whether the identified genes are expressed in all MB neurons or only a fraction?

We agree that the resolution of the HCR signal in the figures was not very high. We have therefore included a supplementary figure (Supplementary Figure_S4) where, for each gene, we show a magnified image of individual cells and 2 alternative sections of the MB cells. The identified genes are expressed in a fraction of MB cells, as more clearly seen in the magnified images, which we now also state in the manuscript.

5. Why was the choice made to focus on unc5 of all the hits?

The reason why we chose *unc-5* is based on the available documentation on its role in synaptic plasticity and its indirect implication in other learning and memory studies. We have updated the manuscript to include this clarification. Thank you for pointing this out.

6. Given all the data on Kenyon cell subtype specificity in memory it is surprising that unc5 knockdown has an effect in all cell types. Why might this be?

The knockdown of *unc-5* showed indeed a phenotype in all the MB cellular subsets, and these results (Fig 5A) are consistent with its effect on each memory phase tested in this study, so 0h, 24h and 48h memory (Fig 3C). To exclude any potential implication linked to the developmental stage, we restricted *unc-5* knockdown to the adult stage by using tub-Gal80ts. The results are now presented in the new Fig4 indicating that unc-5 is involved in LTM-regulatory mechanisms, apart from development. We have updated the manuscript accordingly.

Line 48: Memory consolidation also refers to protein-synthesis independent memories that stabilize following learning. To clarify, I would explicitly state 'protein synthesis-dependent long-term memories.

We have updated the manuscript accordingly.

Line 68-72: I presume these differences in CREB are simply because it serves the same functions in circuits dedicated to different types of memory.

Yes, in addition, CrebB function also relies on the activation of other cofactors which might be differentially available among the circuits. The mentioned paragraph is now updated to include this information. Thanks.

Line 124: Suggest not referring to the control group as Naïve, because the have been trained…simply in a way that does not confer memory.

Contrary to the unpaired group, which undergoes similar training procedures of the experimental paired group (but without associative event), the naïve group was constituted by untreated flies that have never undergone any sensory association.

The lines showing significance between controls and experimental are unclear. Simply showing the star would be clearer.

We have tried to update our plots as the reviewer suggests. However, because in some cases the experimental data are compared to more than one control, we believe that the lines could be actually helpful.

Line 271: Is esn expression very low, or not expressed?

Both the HCR and the single cell data show that a very low percentage of KCs express esn.

Line 349: The statement is a big assumption.

We agree that the mentioned sentence was not totally correct. We have now updated it. Thanks
