# Supplementary material for: Memory phase-specific genes in the Mushroom Bodies identified using CrebB-target DamID
Source: PLoS Genet. 2023 Jun 12;19(6):e1010802. doi: 10.1371/journal.pgen.1010802 (PMC10289670; doi:10.1371/journal.pgen.1010802)
Supplement: Attachment — Submitted filename: Reviewer_Response.docx [file pgen.1010802.s014.docx]

The authors have addressed most of my previous comments. However, I still have one concern regarding the RNAi knockdown efficiency of unc-5 is not very significant in Q-PCR data (see Figure S3), but significant behavioral phenotype was shown when unc-5-RNAi expression in mushroom body (see Figure 2G, 3C, 4D, 5). The author should at least have some discussions/explanations regarding the possibility of these inconsistent results in the manuscript.

Indeed, the qPCR for unc-5 did not show a significant reduction of RNA levels. We have now addressed this in the discussion sections with the following explanation: “Despite having a clear behavioral phenotype, the qPCR does not indicate a significant reduction in *unc-5* levels. One explanation would be that for the behavioral assay *unc-5* was reduced only in the mushroom body affecting specifically the cells required for LTM formation whereas for the qPCR expression of the RNAi was driven with the pan-neuronal elav-Gal4 driver and whole heads were used for RNA extraction. Therefore, non-neuronal tissue might mask or compensate the loss of unc-5 in neurons. For instance, *unc-5* is also expressed in glia cells (76) and is involved in short-range repulsion during motor axon guidance (77). Nonetheless, the fact that we observe the same memory phenotype with three different *unc-5* RNAi-lines targeting non-overlapping regions of the *unc-5* mRNA, strongly supports a specific requirement of Unc-5 in LTM stability.”
